# Association between sodium-glucose cotransporter 2 (SGLT2) inhibitors and lower extremity amputation: A systematic review and meta-analysis

**DOI:** 10.1371/journal.pone.0234065

**Published:** 2020-06-05

**Authors:** James Heyward, Omar Mansour, Lily Olson, Sonal Singh, G. Caleb Alexander

**Affiliations:** 1 Center for Drug Safety and Effectiveness, Johns Hopkins Bloomberg School of Public Health, Baltimore, Maryland, United States of America; 2 Monument Analytics, Baltimore, Maryland, United States of America; 3 Department of Family Medicine and Community Health, University of Massachusetts Medical School, Worcester, Massachusetts, United States of America; 4 Department of Epidemiology, Johns Hopkins Bloomberg School of Public Health, Baltimore, Maryland, United States of America; 5 Division of General Internal Medicine, Johns Hopkins Medicine, Baltimore, Maryland, United States of America; University of Oxford, UNITED KINGDOM

## Abstract

**Background:**

The association between sodium-glucose cotransporter 2 inhibitors (SGLT2i’s) and lower extremity amputation is unclear.

**Purpose:**

To systematically review randomized control trials (RCTs) and observational studies quantifying risk of lower extremity amputations associated with SGLT2i use.

**Data sources and study selection:**

We searched PubMed, EMBASE, Scopus, and the Cochrane Central Register of Controlled Trials from January 2011 to February 2020 for RCTs and observational studies including lower extremity amputation outcomes for individuals with type 2 diabetes mellitus treated with SGLT2i’s vs. alternative treatments or placebo.

**Data extraction and synthesis:**

Two reviewers independently extracted data.

**Main outcomes and measures:**

Our primary outcome was risk of lower limb amputation. Secondary outcomes included peripheral arterial disease, peripheral vascular disease, venous ulcerations, and diabetic foot infections. We also evaluated the risk of bias. We conducted random and fixed effects relative risk meta-analysis of RCTs.

**Results:**

After screening 2,006 studies, 12 RCTs and 18 observational studies were included, of which 7 RCTs and 18 observational studies had at least one event. The random effects meta-analysis of 7 RCTs suggested the absence of a statistically significant association between SGLT2i exposure with evidence of substantial statistical heterogeneity (n = 424/23,716 vs n = 267/18,737 in controls; RR 1.28, CI’s 0.93–1.76; I^2^ = 62.0%; p = 0.12) whereas fixed effects analysis showed an increased risk with statistical heterogeneity (RR 1.27, 1.09–1.48; I^2^ = 62%; p = 0.003). Subgroup analysis of canagliflozin vs placebo showed a statistically significantly increased risk in a fixed effects meta-analysis (n = 2 RCTs, RR 1.59, 1.26–2.01; I^2^ = 88%; p = 0.0001) whereas the meta-analysis of dapagliflozin or empagliflozin (n = 2 RCTs each) and a single RCT for ertugliflozin did not show a significantly increased risk. The findings from observational studies were too heterogeneous to be pooled in a meta-analysis and draw meaningful conclusions. Both randomized and observational studies were of generally good methodological quality.

**Conclusions:**

Overall, there was no consistent evidence of SGLT2i exposure and increased risk of amputation. The increased risk of amputation seen in the large, long-term Canagliflozin Cardiovascular Assessment Study (CANVAS) trial for canagliflozin, and select observational studies, merits continued exploration.

## Introduction

In 2017, 30.3 million individuals in the United States were estimated to have diabetes, increasing their risk for microvascular and macrovascular morbidities [1]. Lifestyle modification and pharmacotherapy can help to prevent these complications by reducing glycemic burden and promoting glycemic control.

Sodium-glucose cotransporter 2 inhibitors (SGLT2i’s) are anti-hyperglycemic agents (AHA) first approved by the U.S. Food Drug Administration (FDA) in 2013 for type 2 diabetes. Unlike other diabetes treatments, SGLT2i’s, including canagliflozin, dapagliflozin, and empagliflozin, inhibit renal glucose reabsorption, increasing glucose excretion and decreasing plasma glucose concentrations. SGLT2i’s work independently of insulin production and offer additional clinical benefits including weight loss [2] and reduced risk of major cardiovascular events, heart failure and, all-cause death [3].

Against these potential benefits, in 2017, the FDA issued a Drug Safety Communication, concluding that canagliflozin causes an increased risk of leg and foot amputation [4]. The FDA based their decision on two clinical trials that found a statistically significantly greater risk of amputation with canagliflozin compared to placebo (6.3 vs 3.4 participants with amputations per 1000 patient-years, hazard ratio (HR) 1.97 95% confidence intervals (CI) 1.41–2.75) [5]. Those trials only studied canagliflozin and were not statistically powered to assess amputations, but evidence from a meta-analysis of randomized trials supported this assertion, finding a statistically significant increase in risk of amputation for SGLT2i’s compared to active controls or placebo (relative risk (RR) 1.44; CI 1.13–1.83) [3].

Despite this evidence, some observational studies have not detected an association [6][7] or have found a lower risk of amputation from SGLT2i’s versus sulfonylureas [8], and the mechanism by which SGLT2i might increase the risk of amputations is unknown [9]. A review of SGLT2i’s limited to randomized controlled trials published between January 2015 and June 2017 noted an increased risk of amputations in one trial [10]. In addition to updating this prior review limited to RCTs on the outcome of amputations, we also included observational studies and evaluated peripheral vascular events.

## Methods

### Systematic review registration

We conducted a systematic review and meta-analysis following a prespecified protocol published in the PROSPERO International Prospective Register of Systematic Reviews [11]. We followed the Preferred Reporting Items for Systematic Reviews and Meta-Analysis (PRISMA) checklist (**S1 Appendix**).

### Data sources and searches

We searched PubMed, EMBASE, Scopus, and the Cochrane Central Register of Controlled Trials (CENTRAL), using combined text and Medical Subject Heading (MeSH) terms on March 13, 2019, and updated our search on February 13, 2020 (**S2 Appendix**). The detailed search strategy including MeSH terms used is published on PROSPERO (ID CRD42019119069) [11]. We included studies published from 2011-present, as the first global approval of a SGLT-2i occurred in 2011. No language restrictions were applied.

### Study selection

We included randomized controlled trials (RCT) and observational studies, including retrospective or prospective cohort studies, case-control, and self-controlled studies. The included studies enrolled subjects 18 years or older with type 2 diabetes receiving SGLT2i’s compared against other AHAs or placebo. Two authors (JH and LO) independently reviewed titles and abstracts of retrieved studies to identify those that potentially met inclusion criteria. Two team members then retrieved and independently assessed the full text of potentially eligible studies. Disagreements about the eligibility of studies were adjudicated by discussion between the two review team members.

### Outcomes extracted

Our primary outcome was risk of lower limb amputation. Secondary outcomes included peripheral arterial disease, peripheral vascular disease, venous ulcerations, and diabetic foot infections. We included studies that reported any of these outcomes as either a primary or secondary outcome with effect estimates such as odds ratios or risk ratios.

### Data collection and analysis

We used duplicate extraction, with two study authors (JH and LO) independently extracting relevant study characteristics and outcomes into a standardized form **(S3 Appendix)**. In all cases, we extracted study setting, study design, recruitment method, sample size, participant demographics, patient inclusion and exclusion criteria, outcomes and times of measurement, and information for assessment of risk of bias. For observational studies, we also extracted total and median person-time observed by treatment group; outcome event rates; adjusted and unadjusted hazard ratios; and demographic characteristics accounted for in propensity-score matching of treatment and comparator groups. For RCTs, we extracted event counts or event rates to generate odds ratios or relative risks.

### Risk of bias assessment

Two independent reviewers (JH and OM) assessed risk of bias based on the methodological quality of the included studies. Risk of bias for the RCTs was assessed using the Cochrane Risk of Bias Tool for Randomized Controlled Trials, which evaluated trials based on the presence or absence of randomization sequence generation, allocation concealment, selective reporting, blinding of participants and personnel, blinding of outcome assessment, incomplete outcome data, and other forms of bias [12]. For observational studies, we used the Newcastle-Ottawa Scale to rate studies on methods of addressing time-varying confounding, baseline confounding, patient selection, classification of outcomes, deviations from the intended intervention, missing data, measurement of outcomes, and selection of reported outcomes [13]. Disagreements about the risk of bias assessment were adjudicated through discussion among the study team. We reached final consensus prior to inclusion.

### Data synthesis and analysis

#### Quantitative synthesis of RCTs

We pooled the results using a random-effects meta-analysis of RCTs, with risk ratios for binary outcomes, and calculated 95% confidence intervals and p-values for each outcome. We also report results using fixed effects which are appropriate when the number of studies is low. We assessed the amount of heterogeneity across the RCTs examined using the I^2^, a measure of the amount of variation in outcomes due to variance in true effect sizes rather than sampling error. Publication bias was assessed using Funnel plots. All analyses were conducted in RevMan 5.3 [14].

We also conducted some subgroup analysis of RCTs. We conducted meta-analysis evaluating the risk of amputations for each individual SGLT2 inhibitor and the meta-analysis of SGLT2 inhibitors vs placebo in the RCTs.

#### Qualitative synthesis of observational studies

We synthesized our findings in narrative form organized by study design, comparison group, and safety outcome. We summarized the results of individual studies, describing event rates as well as risk ratios, odds ratios or adjusted hazard ratios (observational studies) for developing the vascular outcomes of interest. In our narrative synthesis we compared all SGLT2i’s, as well as individual SGLT2i’s, to alternative AHAs.

## Results

### Study selection

**Fig 1:**
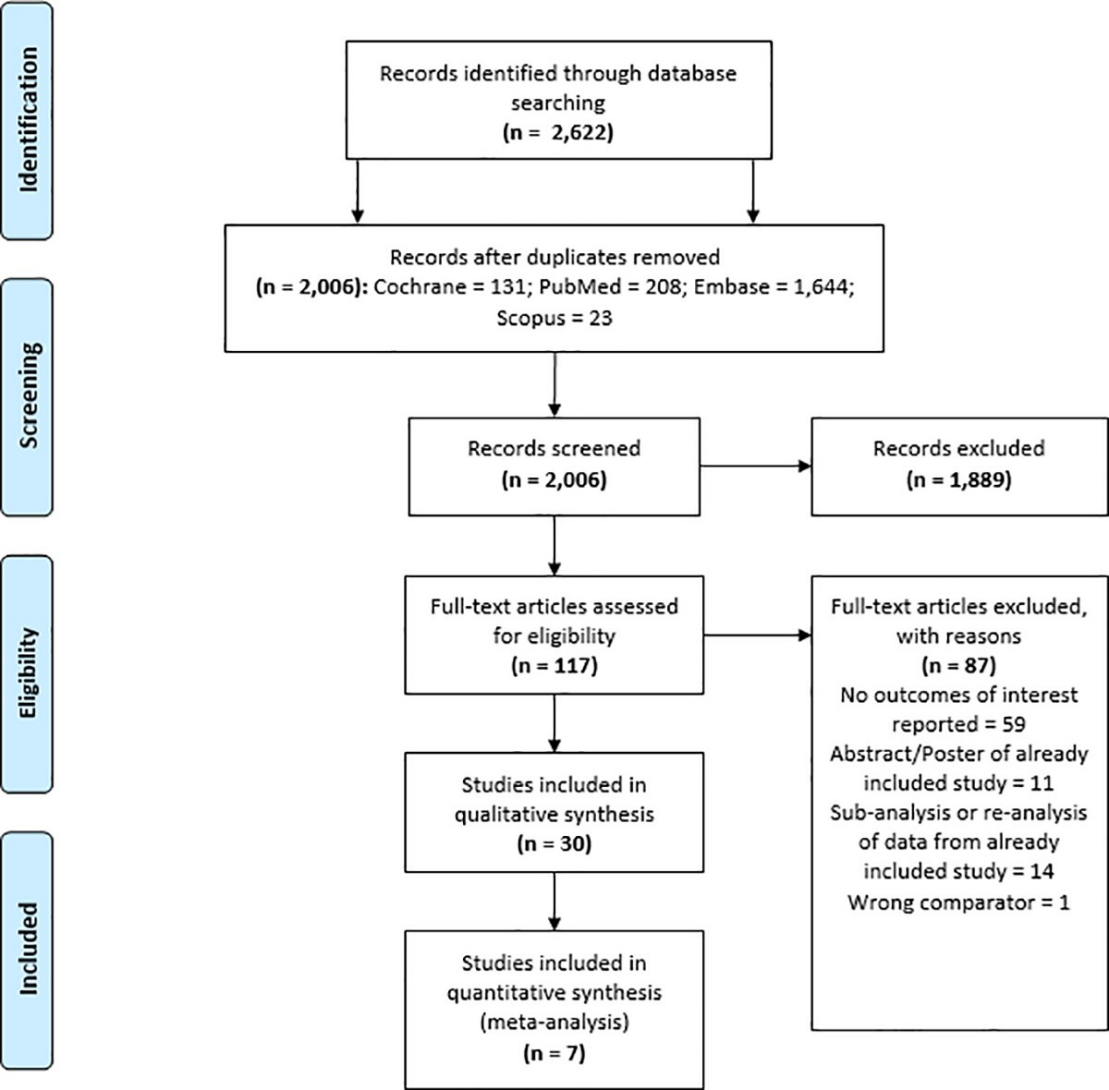
Prisma flow diagram.

The PRISMA flow sheet for studies is shown in **Fig 1**. A total of 2,622 citations were available for screening, and 698 articles were duplicates. One-hundred and seventeen articles remained after title and abstract review, and 30 after full-text screening. We evaluated a total of 12 RCTs (**Table 1**) and 18 observational studies (**Table 2**).

**Table 1:** Characteristics of included randomized controlled trials (N = 12).

Study	Study Duration	Intervention	Intervention Arm	No. of Participants	Age in y (SD)	Smokers, %	CVD History, %	Sex, %	Funding
Fioretto P et al., 2018 [19]	24 weeks	Dapagliflozin 10 mg	Dapagliflozin	160	65.3 (NR)	NR	NR	F- 43.1	AstraZeneca
---	---	---	Placebo	161	66.2 (NR)	NR	NR	F- 43.5	
Hollander P et al., 2018 [25]	52 weeks	Ertugliflozin 5mg or 15 mg as an add-on to metformin ≥1500 mg/day	Ertugliflozin	888	58.5 (9.9)	NR	NR	F- 52.7	Merck
---	---	---	Glimepiride	437	57.8 (9.2)	NR	NR	F- 48.7	
Kashiwagi A et al., 2019 [26]	12, 16, or 24 weeks	Ipragliflozin 50 mg	Ipragliflozin	1209	58.1 (10.3)	NR	NR	F-39.3	Astellas Pharma Co.
---	---		Placebo	796	57.4 (9.9)	NR	NR	F- 42.6	
Kawamori R et al., 2018 [15]	52 weeks	Empagliflozin 10 or 25 mg as add-on to linagliptin in fixed-dose combination	Empagliflozin/ Linagliptin	182	60.0 (9.9)	NR	NR	F- 22.0	Boehringer Ingelheim and Eli Lilly
---	---	---	Placebo/Linagliptin	93	59.8 (10.8)	NR	NR	F- 22.6	
Matthews D et al., 2019 [23]*	188 weeks	Canagliflozin 100 or 300 mg	Canagliflozin	5790	63.2 (8.3)	17.6%	64.4%	F- 35.5	Janssen
---	---		Placebo	4344	63.5 (8.2)	17.9%	66.6%	F- 37.0	
Perkovic V et al. 2019 [22]	2.62 years	Canagliflozin 100 mg	Canagliflozin	2202	62.9 (9.2)	15.5	50.5	F- 34.6	Janssen
---	---	---	Placebo	2199	63.0 (9.2)	13.6	50.3	F- 33.3	
Pollock C et al., 2019 [21]	24 weeks	Dapagliflozin 10 mg	Dapagliflozin	145	64.7 (8.6)	NR	40% cardiac dis, 14% vascular dis	F- 30%	AstraZeneca
---	---	---	Placebo	148	64.7 (8.5)	NR	28% cardiac dis, 16% vascular dis	F- 29%	
Sone H et al., 2019 [17]	52 weeks	Empagliflozin 10 mg	Empagliflozin	86	58.3 (10)	NR	NR	F- 27%	Nippon Boehringer Ingelheim
---	---	---	Placebo	90	59.1 (10.7)	NR	NR	F- 23%	
Terauchi Y et al., 2017 [24]	52 weeks	Tofogliflozin 20 mg	Tofogliflozin-Tofogliflozin	140	59.1 (10.9)	NR	NR	F- 36.4	Sanofi K.K. and Kowa Company
---	---	---	Placebo-Tofogliflozin	70	56.4 (10.0)	NR	NR	F- 31.4	
Wiviott S et al., 2018 [20]	Up to 6 years	Dapagliflozin 10 mg	Dapagliflozin	8582	63.9	NR	NR	F- 36.9	AstraZeneca
---	---	---	Placebo	8578	64.0	NR	NR	F- 37.9	
Yabe D et al., 2019 [18]	Varies (pooled data 15 trials)	Empagliflozin 10 mg	Empagliflozin	724	58.0 (9.9)	NR	NR	F-31%	Boehringer Ingelheim & Eli Lilly & Co. Diabetes Alliance
---	---	---	Placebo	709	58.3 (10.1)	NR	NR	F-37%	
Zinman B et al., 2018 [16]	Up to 4 years	Empagliflozin 10 or 25 mg	Empagliflozin	4687	63.1 (8.6)	NR	NR	F- 28.8	Boehringer Ingelheim and Eli Lilly
---	---	---	Placebo	2333	63.2 (8.8)	NR	NR	F- 28.0	

**NR** not reported; **y** years; **F** female; **dis** disorder

* Demographic data for participants with no amputation

**Table 2:** Characteristics of included observational studies (N = 18).

Source	Treatment Group	Comparison	Participant database	No. of Participants	% CVD	Sex, %	Outcomes	Effect Estimate (Amputation)
Adimadhyam S et al, 2018 [28]	New use of SGLT-2i’s alone	New use of DPP-4i’s alone	Truven MarketScan Commercial Claims	137,012	13.3	F- 45.7	Any amputation after treatment initiation	1.38 (0.83–2.31)
Chang HY et al, 2018 [9]	New use of SGLT-2i’s alone	New use of DPP-4i’s alone, GLP-1’s alone, or sulfonylurea, MET, or TZDs	Truven MarketScan Commercial Claims	973,906	1.72	F- 54.1	LEA, PAD, CLI, osteomyelitis, ulcer	1.50 (0.85–2.67)
Dawwas GK et al, 2019 [8]	New use of SGLT-2i’s alone	New use of sulfonylureas alone or new use of DPP-4i’s alone	Truven MarketScan Commercial Claims	1,072,028	19.6	F- 80.4	CVD, HHF, LEA	0.88 (0.65–1.15)
Fralick M et al., 2019 [36]	New use of canagliflozin	New use of GLP-1 agonists	Adults with T2DM identified using MarketScan, Optum, and Medicare prescription claims databases	321,254	NR	NR	LEA	1.66 (1.33–2.07) (≥65 years) 1.09 (0.89–1.34) (<65 years)
Kaku K et al., 2020 [40]	New use of empagliflozin	None	Adults with T2DM newly initiating empagliflozin treatment	7,618	7.2	F- 36.7	LEA	No comparator
Kashambwa R et al., 2019 [29]	New use of SGLT-2 inhibitors	New use of DPP-4 inhibitors	T2DM patients identified using TriNetX analytics	10,538	NR	NR	Acidosis, acute kidney failure, acute pancreatitis, LLA	0.55 (NR) (Risk Ratio)
McGurnaghan SJ et al., 2019 [42]	New use of dapagliflozin	Never-use of dapagliflozin	Patients identified from a nationwide health and administrative register in Scotland.	238,876	NR	F- 44.3	CVD, DKA, LLA	1.29 (0.71–2.36)
Patorno E et al. 2019 [30]	New use of SGLT-2 inhibitors	New use of GLP-1 agonists	Medicare-insured adults with T2DM	88,358	40.5	F- 54.6	Severe hypoglycemia, bone fractures, LLA, DKA	1.47 (1.07, 2.04)
Patorno E et al, 2019 (2) [41]	New use of empagliflozin	New use of DPP-4 inhibitors	Medicare-insured adults with T2DM	35,078	NR	NR	HHF, ACM, LLA, bone fractures, DKA	1.12 (0.55–2.30)
Paul S et al., 2019 [31]	New use of SGLT-2 inhibitors	New use of GLP-1 agonists, new use of DPP-4 inhibitors, and new use of other antidiabetes drugs	T2DM patients identified using nationally representative primary and ambulatory care EMRs of UK and US	1,844,806	NR	NR	Any amputation, LLA	No between-groups comparison
Pelaez-Bejarano A et al, 2019 [32]	New use of SGLT-2 inhibitors	None	Adults with T2DM	110	NR	NR	LLA	No comparator
Ryan PB et al, 2018 [6]	New use of canagliflozin alone	New use of other SGLT-2i’s (empagliflozin or dapagliflozin), and all non-SGLT2i’s (any DPP‐4i’s, GLP‐1’s, TZDs, sulfonylureas, insulin or other AHAs)	Truven MarketScan Commercial, Medicaid and Medicare Claims; Optum Insight Datamart	1,060,449	30.2	NR	HHF, BKLE	1.01 (0.93–1.10)
Sung J et al., 2018 [33]	Use of SGLT-2 inhibitors	Non-use of SGLT-2 inhibitors	Adults with T2DM attending a foot-wound clinic in a tertiary hospital in Sydney, Australia.	108	NR	F- 27.8	LLA, including minor and major amputations	0.70 (0.29–1.71)
Udell JA et al, 2020 [37]	New use of canagliflozin	New use of non-SGLT-2 inhibitors	Active or retired service members and dependents using Department of Defense data	110,229	100	F- 43.8	ACM, HHF, BKLE amputation	1.44 (0.82, 2.52)
Ueda P et al., 2018 [34]	New use of SGLT-2 inhibitors	New use of GLP1 receptor agonists	Patients identified from nationwide health and administrative registers in Sweden and Denmark.	48,286	NR	F- 40.3	LLA, bone fracture, DKA, AKI, serious UTI, VTE, acute pancreatitis, toe or metatarsal amputation and to major osteoporotic fracture	1.90 (1.25–2.87)
Woo V et al., 2018 [38]	New use of canagliflozin		SGLT-2 naïve adults with T2DM receiving clinical treatment in Canada.	527	NR	F- 40.9	Genital mycotic infections, polyuria, UTE, severe hypoglycemia, volume-related AE, DKA, amputation.	No comparator
Yang JY et al., 2019 [35]	New use of SGLT-2 inhibitors	New use of GLP-1 agonists and new use of sulfonylureas	Commercially insured adults^1^	196,501	NR	F- 44.5	LEA, tissue and bone debridement, PVD, and diabetic foot ulcer.	1.43 (1.01–2.03)
Yuan Z et al, 2018 [39]	New use of canagliflozin	New use of non-SGLT-2i’s (DPP-4i’s, GLP-1’s, TZDs, sulfonylureas, insulin or other AHAs) plus standard of care	Truven MarketScan Commercial Claims	346,190	NR	F- 44.5	BKLE amputation	0.98 (0.68–1.41)

**SGLT2i’s** sodium-glucose cotransporter 2 inhibitors; **CVD** cardiovascular disease, including non‐fatal myocardial infarction or non‐fatal stroke; **GLP-1’s** glucagon-like peptide 1 receptor agonist; **DPP4-i’s** dipeptidyl peptidase 4 inhibitors; **MET** metformin; **TZDs** thiazolidinediones; **PAD** peripheral arterial disease; **CLI** critical limb ischemia; **AHAs** antihyperglylcemic agent; **LEA** lower extremity amputation; **PAD** peripheral arterial disease; **HHF** hospitalization for heart failure; **LLA** lower limb amputation; **DKA** diabetic ketoacidosis; **ACM** all-cause mortality; **AKI** acute kidney injury; **UTI** urinary tract infection; **VTE** venous thromboembolism; **PVD** peripheral vascular disease; **BKLE** below the knee, lower extremity

#### Results of RCTs

*Study design and characteristics*. We included 12 RCTs in our qualitative synthesis. The 12 RCTs included 45,551 participants: 25,593 were randomized to receive an SGLT2 inhibitor, 600 received an alternative treatment, and 19,358 received a placebo (**Table 1**). The trials enrolled from 210 participants to 17,160 participants and ranged in duration from 12 weeks to 8 years. The study design and characteristics of included RCTs are shown in **Table 1**. Four studies examined empagliflozin [15][16][17][18], three studies examined dapagliflozin [19][20][21], two studies examined canagliflozin [22][23], and 1 each examined topogliflozin [24], ertugliflozin [25], and ipragliflozin [26]. Eleven studies used a placebo control and the ertugliflozin study used glimepiride as the control. Five studies, two examining empagliflozin and 1 each examining dapagliflozin, ipragliflozin, and topogliflozin, reported 0 amputation events in both arms and thus seven studies were available for meta-analysis.

### Risk of bias of RCTs

Overall, there was little to no evidence of major bias in the included RCTs (**S1 Fig**). However, reporting of study methodology was often incomplete. For example, the method of randomizing participants was only reported in 7 of 12 RCTs. In cases in which randomization method was not specified, we assumed truly random allocation based on its fundamental and universally recognized importance (“probable yes”). Concealment of the allocation sequence was never reported. Baseline demographic characteristics were uniformly well presented and loss to follow-up was relatively low.

### Meta-analysis of the risk of amputation with SGLT2 inhibitors vs controls in RCTs

**Fig 2:**
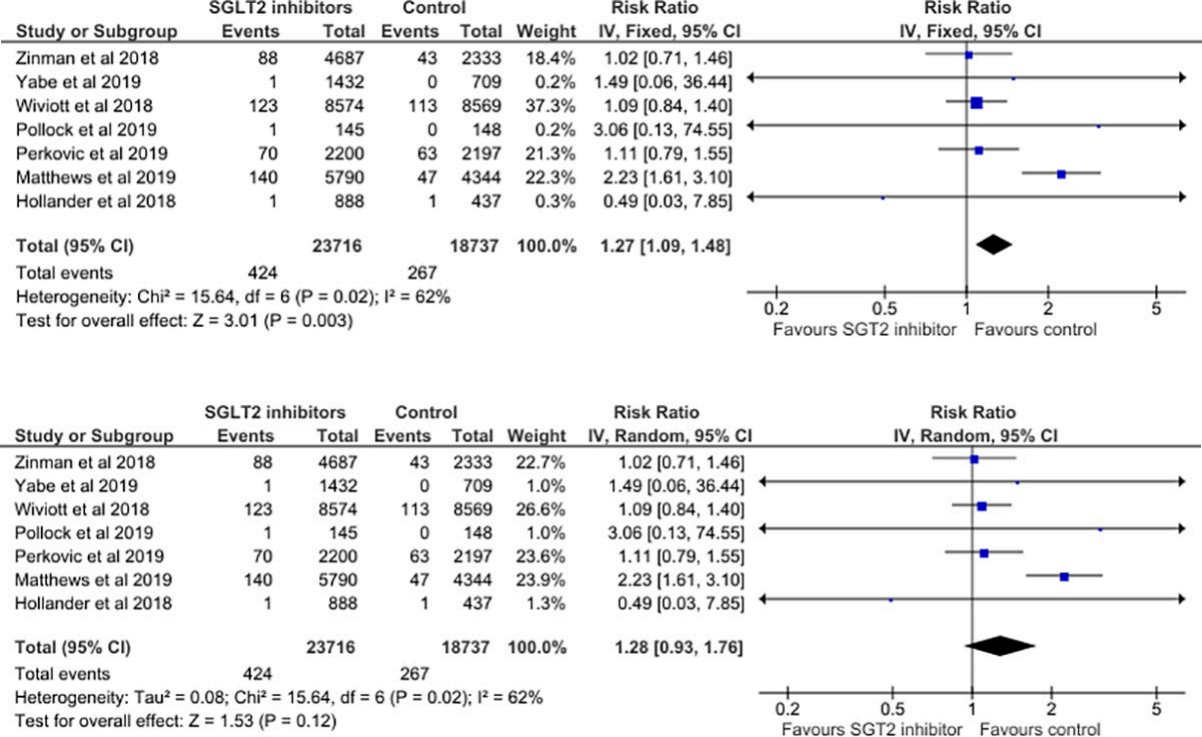
A. Fixed-effects meta-analysis- SGLT2i’s vs. placebo/glimepiride. B. Random-effects meta-analysis- SGLT2i’s vs. placebo/glimepiride.

**Fig 2** depicts the meta-analysis examining the association between SGLT2i exposure and lower extremity amputation based on 7 RCTs. The random effects meta-analysis of 7 RCTs suggested the absence of a statistically significant association between SGLT2i exposure with evidence of substantial statistical heterogeneity (n = 424/23,716 vs n = 267/18,737 in controls; RR 1.28, CI’s 0.93–1.76; I^2^ = 62.0%; p = 0.12) whereas fixed effects analysis showed an increased risk with statistical heterogeneity (RR 1.27, 1.09–1.48; I^2^ = 62%; p = 0.003).

### Subgroup analysis

Subgroup analysis of canagliflozin vs placebo showed a statistically significantly increased risk in a fixed effects meta-analysis (n = 2 RCTs, RR 1.59, 1.26–2.01; I^2^ = 88%; p = 0.0001) whereas the meta-analysis of dapagliflozin or empagliflozin (n = 2 RCTs each) did not show a significantly increased risk (**Table 3**). Although we present both the results for the fixed and random effect meta-analysis of dapagliflozin, empagliflozin and canagliflozin vs placebo, the fixed effects results are considered most appropriate when number of studies is low (n = 2 for each subgroup).

**Table 3:** Subgroup analysis of risk of amputation among individual SGLT2i’s.

	No. of studies	No. of events in SGLT-2i arm/ Total no. of participants	No. of events in control/ Total no. of participants	IV weighted RR Random effects; I^2,^ %	IV weighted RR Fixed effects; I^2,^ %
Empagliflozin vs placebo (Yabe et al [21], Zinman et al [16])	2	89/6119	43/3042	1.02 [0.71–1.47]; I^2^ = 0%	1.02 [0.71–1.47]; I^2^ = 0%
Dapagliflozin vs control (Wiviott et al [20], Pollock et al [21])	2	124/8719	113/8717	1.09 [0.85–1.41]; I^2^ = 0%	1.09 [0.85–1.41]; I^2^ = 0%
Canagliflozin vs placebo (Perkovic et al [23], Matthews et al [24])	2	210/7990	110/6541	1.58 [0.79–3.13]; I^2^ = 88%	1.59 [1.26–2.01]; I^2^ = 88%
**SGLT2 inhibitors vs placebo**					
SGLT2 inhibitors vs placebo (Yabe et al [18], Zinman et al [16], Wiviott et al [20], Pollock et al [21], Perkovic et al [22], Matthews et al [23])	6	422/22828	267/18300	1.27 [0.91–1.77]; I^2^ = 68%	1.27 [1.08–1.48]; I^2^ = 68%

**SGLT2i’s** sodium-glucose cotransporter 2 inhibitors; **No.** number; **IV** inverse variance; **RR** relative risk

Only one study evaluated the risk of SGLT2i vs active comparator and reported no risk [27]. The meta-analysis of remaining 6 placebo-controlled studies showed no significant increased risk of amputations associated with SGLT2 inhibitors in a random effects meta-analysis (RR 1.27,0.91–1.77; I^2^ = 68%).

#### Results of observational studies

*Study characteristics*. Of the 18 observational studies included, 15 were retrospective cohort studies, 2 were prospective cohort studies, and 1 was a case-control study. The studies used claims records of more than 6.4 million individuals; 860,120 (13.3%) of them were classified as new users of SGLT2i’s. The study design and characteristics of included observational studies are shown in **Table 2**.

### Risk of bias of observational studies

The included observational studies were of generally good quality (**S2 Fig**), although 3 lacked a control arm and 2 did not adjust for potential baseline confounding between exposed and non-exposed individuals. The included studies were broadly similar in terms of ascertainment of exposure and assessment of outcomes (i.e. electronic health claims and administrative codes), but varied when it came to representativeness of the exposed cohort. In addition, only 8 studies explicitly assessed and excluded amputation at baseline. Most studies adjusted for confounding; 12 used propensity score matching on a broad array of baseline characteristics, and one matched patients based on a predefined list of baseline factors. In all studies, the length of follow-up observation was relatively sufficient to assess the outcomes of interest.

### Qualitative synthesis of observational studies

Eleven examined SGLT2i’s as a class [28][9][8][29][30][31][32][33][34][35][36], 4 examined canagliflozin alone [6][37][38][39], 2 examined empagliflozin alone [46][47], and 1 examined dapagliflozin alone [42]. Comparator products varied significantly among studies, with 6 each using DPP-4 inhibitors (DPP-4i) and GLP-1 agonists (GLP-1a); 3 using all non-SGLT2i’s AHAs combined; 2 using sulfonylureas; 2 using non-use of SGLT2i’s, and 3 with no comparison group and 1 with no testing of inter-group differences. Several studies included multiple comparator classes.

Across all 19 reported active-comparator analyses in the 15 studies with a comparator, 6 reported a decreased risk of amputation among SGLT2i users, though level of adjustment, statistical significance, and comparator varied. The adjusted effect estimates of 13 analyses showed an increased risk of amputation; again, significance and comparator varied. Inter-study heterogeneity prevented any meta-analysis of analyses comparing any SGLT2i therapy to a specific comparator.

### Secondary outcomes

#### Randomized controlled trials

None of the included RCTs measured any of the secondary outcomes of interest.

#### Observational studies

One included study reported on peripheral vascular disease and venous ulcerations, reporting three separate analyses for each [9]. The adjusted HR for incident peripheral vascular disease comparing SGLT2’i vs. DPP-4i’s, GLP-1a’s, and all non-SGLT2i AHAs, respectively, were 0.88 (95% CI 0.79–0.96), 0.95 (95% CI 0.84–1.07), and 1.11 95% CI (1.02–1.22), and for venous ulceration 1.12 (95% CI 0.91–1.39), 0.97 (95% CI 0.75–1.26), and 1.34 (95% CI 1.10–1.61). No studies examined peripheral arterial disease or diabetic foot infections.

## Discussion

More than seven years after their market debut in the United States, questions remain regarding the potential association between SLGT2i’s and lower extremity amputation. In this systematic review and meta-analysis, amputation risk varied widely among the studies that were synthesized; data from randomized studies comparing five different SGLT2i’s to placebo or glimepiride indicated a statistically significant elevated amputation risk in one large, long-term trial for canagliflozin only, and a non-significant association overall. Subgroup analysis showed a statistically significantly increased risk for canagliflozin alone.

Among observational data, study heterogeneity and potential confounding prevented the conduct of meta-analysis, but we found that two thirds of analyses comparing SGLT-2i products against GLP-1a’s, DPP-4i’s, sulfonylureas and other AHA’s reported an elevated risk of amputation among SGLT-2i’s, though the effect was rarely statistically significant. Taken together, the preponderance of evidence suggested no consistent evidence of association between SGLT2i exposure and increased risk of amputation among adults with type 2 diabetes, though the risk associated with canagliflozin exposure bears further scrutiny. These findings are important given how commonly SGLT2i’s are prescribed, as well as ongoing questions regarding their optimal role in the treatment of a common and costly chronic disease.

Our review underscores the heterogeneous literature regarding SGLT2i’s and adverse events such as lower extremity amputation. The studies were diverse with respect to study design, duration, reference product, comparator, and statistical and reporting methods. Although we were unable to combine all studies for meta-analysis due to heterogeneity, we conducted several meta-analyses that included comparisons of the SGLT2i’s class or individual members of that class against placebo, but not GLP-1a’s, DPP-4i’s, sulfonylureas or aggregated non-SGLT2i therapies, to minimize the possibility of confounding by indication and disease severity. In our meta-analyses there were differences between the models due to the effect of smaller studies, which have relatively greater weight in random than fixed effects models. Marked heterogeneity in the included studies argued for use of random effects as primary results [43], except when the number of studies is low, although these may not always provide a conservative estimate of risk [44].

Our findings bear similarities and differences to other meta-analysis on this topic. In additional to the previously referenced meta-analysis [10], two recent meta-analyses of RCTs published in May and September 2019, respectively, found a non-significant increased risk of amputation among three large studies assessing canagliflozin, dapagliflozin, and empagliflozin [45][46]. The overall finding of non-significance in these studies mirrors our own, while in contrast, we were able to meta-analyze the results for three individual products, and our findings were also supplemented by the inclusion of observational studies that similarly were inconclusive in aggregate but also were suggestive an elevated risk for canagliflozin.

One important consideration is whether the heterogeneous effect seen in our study may be limited to a particular drug and not a class effect [47]. We found that among discrete SGLT-2i products meta-analyzed using RCT data, only canagliflozin carried a statistically significant elevated risk of amputation compared with placebo treatment. This is important, and bears further scrutiny in future investigations. Similarly, if the risk is limited to those with high baseline CVD such as those enrolled in the Canagliflozin Cardiovascular Assessment Study (CANVAS) program, an individual participant data meta-analysis may provide further information.

Studies that evaluate the biological mechanisms that could account for any such risk of SGLT2i’s are also needed. For example, it is unclear whether any increased risk of amputation, should it be present, is due to the diuretic effect of SGLT2i’s; some studies have suggested that diuretics may increase the risk of amputations in patients with type 2 diabetes [48][49].

The potential risks of SGLT2i’s must be balanced with their potential benefits, including improved glycemic control and reduced rate of major adverse cardiovascular events (MACE) as demonstrated for empagliflozin in the BI 10773 [Empagliflozin] Cardiovascular Outcome Event Trial in Type 2 Diabetes Mellitus Patients (EMPA-REG OUTCOME) trial [16], for canagliflozin in the CANVAS program, which included both the CANVAS and CANVAS-RENAL trials, and for dapagliflozin in the Dapagliflozin Effect on Cardiovascular Events–Thrombolysis in Myocardial Infarction (DECLARE-TIMI) trial [20]. Data from these trials suggest significant cardiovascular benefits for individuals with pre-existing CVD. This evidence has led FDA to expand the label for empagliflozin and canagliflozin for use to lower cardiovascular risk in patients with type 2 diabetes and cardiovascular disease [50], and it has shaped practice guidelines that underscore the selection of agents, especially for those with cardiovascular disease, based on their proven ability to reduce major adverse cardiovascular events and/or cardiovascular mortality [51][52]. As with other pharmacologic treatments for diabetes, these features of SGLT2i’s underscore the importance of individualized selection of therapies based on factors including regimen effectiveness, adverse event profile, formulation, therapeutic complexity, cost, and patient preference.

Despite its rigor, our study has limitations, some reflecting features of the individual studies that we examined. Although RCTs are the principal means of establishing the efficacy of drugs, they may have limited statistical power to detect infrequent adverse events, such as amputations in real-world patients, which occurs at a rate of 5.0 per 1,000 in individuals with type 2 diabetes [53]. Thus these RCTs are quite susceptible to type 2 error [54]. None of the included trials pre-specified lower extremity amputations as an outcome of ascertainment, rather these data were collected as adverse events, which may result in misclassification of outcomes; any such misclassification is likely to be non-differential and would bias the results towards the null. In addition, we pooled analyses with limited clinical information on patients’ baseline cardiovascular risks, and no individual-level patient data was available to carry out prespecified subgroup analysis based on preexisting CVD status. Also, the studies reflected considerable heterogeneity, preventing more precise estimates of the associations of interest. Our study focused on the peer-reviewed literature, and it is possible that trial registries, grey literature, or other non-peer reviewed, publicly available information might contain additional data relevant to the association between SGLT2i’s, lower extremity amputation, and other cardiovascular events.

## Conclusion

Given the elevated incidence of cardiovascular disease among individuals with type 2 diabetes, the cardiovascular risks, and benefits, of pharmacologic treatments for diabetes are of perennial interest and concern. Despite reproducible, well-controlled evidence of significant reductions in major adverse cardiovascular events associated with SGLT2i’s compared with placebo, the association between SGLT2i’s and lower extremity amputation has been much less clear. In this systematic review and meta-analysis, we found no consistent evidence of SGLT2i exposure and increased risk of amputation. The increased risk of amputation observed in the large, long-term CANVAS trial with canagliflozin, and select observational studies, merits further exploration.

## Supporting information

S1 Appendix Preferred reporting items for systematic reviews and meta-analysis (PRISMA) checklist. (DOCX)

S2 Appendix Search strategy. (DOCX)

S3 Appendix Data extraction form. (DOCX)

S4 Appendix Event counts from included rcts. (DOCX)

S5 Appendix Risk of bias assessment for individual randomized controlled trials according to the cochrane collaboration tool (N = 12 studies). (DOCX)

S6 Appendix Risk of bias assessment of observational studies reporting on SGLT2-inhibitors and lower extremity amputation using the newcastle-ottawa scale (N = 18 studies). (DOCX)

S1 Fig Risk of bias assessment of randomized controlled trials reporting on SGLT2-inhibitors and lower extremity amputation using the cochrane risk of bias tool (N = 12 studies). (TIFF)

S2 Fig Risk of bias assessment of observational studies reporting on SGLT2-inhibitors and lower extremity amputation using the newcastle-ottawa scale (N = 18 studies). (TIFF)
