# Supplementary material for: Association between sodium-glucose cotransporter 2 (SGLT2) inhibitors and lower extremity amputation: A systematic review and meta-analysis
Source: PLoS One. 2020 Jun 5;15(6):e0234065. doi: 10.1371/journal.pone.0234065 (PMC7274434; doi:10.1371/journal.pone.0234065)
Supplement: S2 Appendix — (DOCX) [file pone.0234065.s002.docx]

**APPENDIX 2. SEARCH STRATEGY**

**Revised SGLT2 Inhibitors and Amputation**

**November 29, 2018**

| **Database** | **Results** |
| --- | --- |
| **PubMed** | 127 |
| **Embase**—SGLT2i plus amputation terms | 188 |
| **Embase**—plus amputation and two terms for serious adverse events | 286 |
| **Embase**—all plus peripheral artery/vascular diseases | 1,162 |
| **Scopus** | 204 |
| **Cochrane Trials** (43 total hits with reviews and protocols) | 34 |

**PubMed**

"Sodium-Glucose Transporter 2/antagonists and inhibitors"[mesh] OR "Sodium-Glucose Transporter 2 Inhibitors" [Pharmacological Action] OR "Sodium-Glucose Transporter 2 Inhibitors"[Mesh] OR canagliflozin[mesh] OR sodium-glucose transporter 2 inhibit*[tw] OR sodium-glucose transporter 2 antagonist*[tw] OR sodium dependent glucose cotransporter 2 inhibit* [tw] OR sodium dependent glucose co-transporter 2 inhibit* [tw] OR sodium glucose cotransporter 2 inhibit* [tw] OR sodium glucose co-transporter 2 inhibit* [tw] OR SGLT2 inhibit* [tw] OR SGLT2 antagonist*[tw] OR SGLT2i[tw] OR canagliflozin [tw] OR atigliflozin [tw] OR bexaglifozin [tw] OR “bi 44847” [tw] OR canagliflozin [tw] OR dapagliflozin [tw] OR empagliflozin [tw] OR ertugliflozin [tw] OR ipragliflozin [tw] OR licogliflozin [tw] OR luseogliflozin [tw] OR mizagliflozin [tw] OR “remogliflozin etabonate” [tw] OR “sergliflozin etabonate” [tw] OR sotagliflozin [tw] OR tofogliflozin [tw] OR gliflozin* [tw] OR empagliflozin [Supplementary Concept] OR ipragliflozin [Supplementary Concept] OR remogliflozin etabonate [Supplementary Concept] OR sergliflozin etabonate [Supplementary Concept]

**3,237 hits**

amputation[mesh] OR amputat*[tw] OR diabetic foot[mesh] OR ((limb* [tw] OR leg[mesh] OR leg[tw] OR legs[tw] OR foot[mesh] OR foot[tw] OR feet[tw] OR lower extremit*[tw]) AND (remov*[tw] OR resect*[tw] OR truncat*[tw] OR excis*[tw] OR diabetic[tw])) OR (venous[tw] AND (ulcer[mesh] OR ulcer*[tw])) OR bone health[tw] OR bone metabolism[tw] OR "Peripheral Arterial Disease"[Mesh] OR "Peripheral Vascular Diseases"[Mesh] OR Peripheral Arterial Disease* [tw] OR Peripheral Vascular Disease*[tw] OR serious adverse event*[tw] OR serious adverse reaction*[tw]

**185,056 hits**

**Terms tried in PubMed and rejected as too broad:**

Diabetes Complications [mesh]

((serious[tw] OR major[tw]) AND ("Drug-Related Side Effects and Adverse Reactions"[Mesh] OR adverse effects[sh]))

**Embase**

**(Lines have been reorganized to read more easily. Line numbers from original search preserved, so they are not always in order. Full search as run available but even harder to read.)**

#1. 'sodium glucose cotransporter 2 inhibitor'/exp 6,984 Emtree term

#3. 'sodium dependent glucose cotransporter 2 5,511 Keywords for SGLT2is

inhibit*':ti,ab,kw OR 'sodium dependent glucose

co-transporter 2 inhibit*':ti,ab,kw OR 'sodium

glucose cotransporter 2 inhibit*':ti,ab,kw OR

'sodium glucose co-transporter 2

inhibit*':ti,ab,kw OR 'sodium-glucose transporter

2 inhibit*':ti,ab,kw OR 'sodium-glucose

transporter 2 antagonist*':ti,ab,kw OR 'sglt2

inhibitor*':ti,ab,kw OR 'sglt2

antagonist*':ti,ab,kw OR canagliflozin:ti,ab,kw

OR atigliflozin:ti,ab,kw OR bexaglifozin:ti,ab,kw

OR 'bi 44847':ti,ab,kw OR dapagliflozin:ti,ab,kw

OR empagliflozin:ti,ab,kw OR

ertugliflozin:ti,ab,kw OR ipragliflozin:ti,ab,kw

OR licogliflozin:ti,ab,kw OR

luseogliflozin:ti,ab,kw OR mizagliflozin:ti,ab,kw

OR 'remogliflozin etabonate':ti,ab,kw OR

'sergliflozin etabonate':ti,ab,kw OR

sotagliflozin:ti,ab,kw OR tofogliflozin:ti,ab,kw

OR gliflozin*:ti,ab,kw

#4. '842133 18 0':rn OR '928672 86 0':rn OR '647834 4,456 Registry numbers

15 9':rn OR '1118567 05 7':rn OR '461432 26 8':rn

OR '864070 44 0':rn OR '1210344 57 2':rn OR

'761423 87 4':rn OR '1291094 73 9':rn OR '898537

18 3':rn OR '666843 10 3':rn OR '442201 24 3':rn

OR '408504 26 7':rn OR '1018899 04 1':rn OR

'1201913 82 7':rn OR '903565 83 3':rn

#5. #3 OR #4 6,583

**#6. #1 OR #5 7,429 (All SGLT2i)**

#7. 'limb amputation'/exp OR ('lower limb'/exp AND 38,060 Emtree terms

'amputation'/exp) OR 'foot amputation'/exp OR

'diabetic foot'/exp

#8. amputat*:ti,ab,kw OR reamputat*:ti,ab,kw OR 61,872 core keywords

'diabetic foot':ti,ab,kw

#9. 'venous ulcer*':ti,ab,kw OR 'bone 28,936

health':ti,ab,kw OR 'bone metabolism':ti,ab,kw

#18. (limb*:ti,ab,kw OR leg*:ti,ab,kw OR foot:ti,ab,kw 11,936

OR feet:ti,ab,kw OR lower) AND extremit*:ti,ab,kw

AND (remov*:ti,ab,kw OR resect*:ti,ab,kw OR

truncat*:ti,ab,kw OR excis*:ti,ab,kw OR

diabetic:ti,ab,kw)

**#19. #7 OR #8 OR #9 OR #18 110,629 (amputation)**

**#20. #6 AND #19 188 (SGLT2i and amputation)**

#12. 'serious adverse event*':ti,ab,kw OR 'serious 33,315

adverse effect*':ti,ab,kw

#21. #12 OR #19 143,765

**#22. #6 AND #21 286**

#11. 'peripheral occlusive artery disease'/exp OR 1,855,706

'peripheral vascular disease'/exp OR 'peripheral

occlusive artery disease*':ti,ab,kw OR

'peripheral artery disease*':ti,ab,kw OR

'peripheral vascular disease*':ti,ab,kw

#23. #11 OR #21 1,972,751

**#24. #6 AND #23 1,162 (SGLT2i/all outcome terms)**

**Scopus**

TITLE-ABS-KEY(("Sodium-Glucose Transporter 2" AND inhibit*) OR ("sodium dependent glucose cotransporter 2" AND inhibit*) OR "SGLT2 inhibit*" OR SGLT2i OR atigliflozin OR bexaglifozin OR "bi 44847" OR canagliflozin OR dapagliflozin OR empagliflozin OR ertugliflozin OR gliflozin OR ipragliflozin OR licogliflozin OR luseogliflozin OR mizagliflozin OR “remogliflozin etabonate” OR “sergliflozin etabonate” OR sotagliflozin OR tofogliflozin "sodium dependent glucose cotransporter 2 inhibitor*" OR "sodium dependent glucose co-transporter 2 inhibit*" OR "sodium glucose co-transporter 2 inhibit*" OR "sodium glucose co-transporter 2 inhibit*")

**4,448 hits**

TITLE-ABS-KEY(amputat* OR ((limb* OR leg OR leg OR legs OR foot OR foot OR feet OR lower extremit*) AND (remov* OR resect* OR truncat* OR excis* OR diabetic)) OR “venous ulcer*” OR “bone health” OR “bone metabolism” OR “Peripheral Arterial Disease*” OR “Peripheral Vascular Disease*” OR “serious adverse event*” OR “serious adverse reaction*”)

**161,097 hits**

**Cochrane**

ID Search Hits

#1 MeSH descriptor: [Sodium-Glucose Transporter 2] explode all trees and with qualifier(s): [antagonists & inhibitors - AI] 153

#2 'sodium dependent glucose cotransporter 2 inhibit* OR 'sodium glucose cotransporter 2 inhibit*' OR 'sodium glucose co-transporter 2 inhibit*' 'sodium-glucose transporter 2 inhibit*' OR 'sodium-glucose transporter 2 inhibit*' OR 'sodium-glucose transporter 2 antagonist*' OR 'sglt2 inhibitor*' OR SGLT2i OR 'sglt2 antagonist*' OR canagliflozin OR atigliflozin OR bexaglifozin OR 'bi 44847' OR dapagliflozin OR empagliflozin OR ertugliflozin OR ipragliflozin OR licogliflozin OR luseogliflozin OR mizagliflozin OR 'remogliflozin etabonate' OR 'sergliflozin etabonate' OR sotagliflozin OR tofogliflozin OR gliflozin* 1640

#3 #1 OR #2 1640

#4 MeSH descriptor: [Amputation] explode all trees 398

#5 MeSH descriptor: [Diabetic Foot] explode all trees 788

#6 amputat* OR reamputat* OR re-amputat* OR ((limb* OR leg OR leg OR legs OR foot OR foot OR feet OR lower extremit*) AND (remov* OR resect* OR truncat* OR excis* OR diabetic)) OR “venous ulcer*” OR “bone health” OR “bone metabolism” OR “Peripheral Arterial Disease*” OR “Peripheral Vascular Disease*” OR “serious adverse event*” OR “serious adverse reaction*” 14114

#7 #4 OR #5 OR #6 14116

#8 #3 AND #7 43
