# Supplementary material for: Association between sodium-glucose cotransporter 2 (SGLT2) inhibitors and lower extremity amputation: A systematic review and meta-analysis
Source: PLoS One. 2020 Jun 5;15(6):e0234065. doi: 10.1371/journal.pone.0234065 (PMC7274434; doi:10.1371/journal.pone.0234065)
Supplement: S3 Appendix — (DOCX) [file pone.0234065.s003.docx]

**APPENDIX 3A. DATA EXTRACTION FORM- RCTS**

**APPENDIX 3B. DATA EXTRACTION FORM- OBSERVATIONAL**
