# Supplementary material for: Association between sodium-glucose cotransporter 2 (SGLT2) inhibitors and lower extremity amputation: A systematic review and meta-analysis
Source: PLoS One. 2020 Jun 5;15(6):e0234065. doi: 10.1371/journal.pone.0234065 (PMC7274434; doi:10.1371/journal.pone.0234065)
Supplement: S4 Appendix — (DOCX) [file pone.0234065.s004.docx]

**APPENDIX 4.** **EVENT COUNTS FROM INCLUDED RANDOMIZED CONTROLLED TRIALS (N = 12).**

| Study Authors | Treatment Arm | Control Arm | Number of  Participants | | Number of Foot and  Leg Amputation Events | |
| --- | --- | --- | --- | --- | --- | --- |
|  |  |  | Treatment | Control | Treatment | Control |
| Fioretto et al. | Dapagliflozin | Placebo | 160 | 161 | 0 | 0 |
| Kawamori et al. | Empagliflozin/Linagliptin | Placebo/Linagliptin | 182 | 93 | 0 | 0 |
| Zinman et al. | Empagliflozin | Placebo | 4,687 | 2,333 | 88 | 43 |
| Terauchi et al. | Topogliflozin-Topogliflozin | Placebo-Topogliflozin | 140 | 68 | 0 | 0 |
| Wiviott et al. | Dapagliflozin | Placebo | 8,574 | 8,569 | 123 | 113 |
| Hollander et al. | Ertuglifozin | Glimepiride | 888 | 437 | 1 | 1 |
| Perkovic et al. | Canagliflozin | Placebo | 2,200 | 2,197 | 70 | 63 |
| Kashiwagi et al. | Ipragliflozin | Placebo | 1,209 | 796 | 0 | 0 |
| Matthews et al. | Canagliflozin | Placebo | 5,790 | 4,344 | 140 | 47 |
| Pollock et al. | Dapagliflozin | Placebo | 145 | 148 | 1 | 0 |
| Sone et al. | Empagliflozin | Placebo | 179 | 90 | 0 | 0 |
| Yabe et al. | Empafliflozin | Placebo | 1,432 | 709 | 1 | 0 |

**PY** person-years
