# Supplementary material for: Association between sodium-glucose cotransporter 2 (SGLT2) inhibitors and lower extremity amputation: A systematic review and meta-analysis
Source: PLoS One. 2020 Jun 5;15(6):e0234065. doi: 10.1371/journal.pone.0234065 (PMC7274434; doi:10.1371/journal.pone.0234065)
Supplement: S5 Appendix — (DOCX) [file pone.0234065.s005.docx]

**APPENDIX 5. RISK OF BIAS ASSESSMENT FOR INDIVIDUAL RANDOMIZED CONTROLLED TRIALS ACCORDING TO THE COCHRANE COLLABORATION TOOL (N = 12)**

| **Domain** | Zinman 2015 | Terauchi 2017 | Kawamori 2018 | Hollander 2018 | Fioretto 2018 | Yabe 2019 | Wiviott 2019 | Sone 2019 | Pollock 2019 | Perkovic 2019 | Matthews 2019 | Kashiwagi 2019 |
| --- | --- | --- | --- | --- | --- | --- | --- | --- | --- | --- | --- | --- |
| Randomization process |  |  |  |  |  |  |  |  |  |  |  |  |
| Deviations from intended interventions |  |  |  |  |  |  |  |  |  |  |  |  |
| Missing outcome data |  |  |  |  |  |  |  |  |  |  |  |  |
| Measurement of the outcome |  |  |  |  |  |  |  |  |  |  |  |  |
| Selection of the reported result |  |  |  |  |  |  |  |  |  |  |  |  |
| **Overall** |  |  |  |  |  |  |  |  |  |  |  |  |
| = Low risk; = Some concerns;  = High risk. | | | | | | | | | | | | |
