# Supplementary material for: Association between sodium-glucose cotransporter 2 (SGLT2) inhibitors and lower extremity amputation: A systematic review and meta-analysis
Source: PLoS One. 2020 Jun 5;15(6):e0234065. doi: 10.1371/journal.pone.0234065 (PMC7274434; doi:10.1371/journal.pone.0234065)
Supplement: S6 Appendix — (DOCX) [file pone.0234065.s006.docx]

**APPENDIX 6. RISK OF BIAS ASSESSMENT OF OBSERVATIONAL STUDIES REPORTING ON SGLT2-INHIBITORS AND LOWER EXTREMITY AMPUTATION USING THE Newcastle-Ottawa SCALE (N = 18 STUDIES)**

| **Study** | **Item** | **Assessment** |
| --- | --- | --- |
| **Chang HY et al.** |  |  |
| ***Selection*** | Representativeness of the exposed cohort | Somewhat representative |
|  | Selection of the non-exposed cohort | Drawn from the same community as the exposed cohort |
|  | Ascertainment of exposure | Secure record (e.g., surgical records) |
|  | Demonstration that outcome of interest was not present at start of study | Yes |
| ***Comparability*** | Comparability of cohorts on the basis of the design or analysis | Study controls for potential confounders using propensity scores to compare outcomes between treatment arms |
| ***Outcome*** | Assessment of outcome | Record linkage |
|  | Was follow-up long enough for outcomes to occur | Yes |
|  | Adequacy of follow up of cohorts | Subjects lost to follow up unlikely to introduce bias (10%) - small number lost |
| ***Method of adjustment*** | - | Propensity score matching |
| **Dawwas GK et al.** |  |  |
| **Selection** | Representativeness of the exposed cohort | Somewhat representative |
|  | Selection of the non-exposed cohort | Drawn from the same community as the exposed cohort |
|  | Ascertainment of exposure | Secure record (e.g., surgical records) |
|  | Demonstration that outcome of interest was not present at start of study | Yes |
| **Comparability** | Comparability of cohorts on the basis of the design or analysis | Study controls for potential confounders using propensity scores to compare outcomes between treatment arms |
| **Outcome** | Assessment of outcome | Record linkage |
|  | Was follow-up long enough for outcomes to occur | Yes |
|  | Adequacy of follow up of cohorts | Subjects lost to follow up unlikely to introduce bias (10%) - small number lost |
| **Method of adjustment** | - | Propensity score matching |
| **Adimadhyam S et al.** |  |  |
| **Selection** | Representativeness of the exposed cohort | Somewhat representative |
|  | Selection of the non-exposed cohort | Drawn from the same community as the exposed cohort |
|  | Ascertainment of exposure | Secure record (e.g., surgical records) |
|  | Demonstration that outcome of interest was not present at start of study | Yes |
| **Comparability** | Comparability of cohorts on the basis of the design or analysis | Study controls for potential confounders using propensity scores to compare outcomes between treatment arms |
| **Outcome** | Assessment of outcome | Record linkage |
|  | Was follow-up long enough for outcomes to occur | Yes |
|  | Adequacy of follow up of cohorts | Subjects lost to follow up unlikely to introduce bias (10%) - small number lost |
| **Method of adjustment** | - | Propensity score matching |
| **Ryan PB et al.** |  |  |
| ***Selection*** | Representativeness of the exposed cohort | Truly representative |
|  | Selection of the non-exposed cohort | Drawn from the same community as the exposed cohort |
|  | Ascertainment of exposure | Secure record (e.g., surgical records) |
|  | Demonstration that outcome of interest was not present at start of study | Yes |
| ***Comparability*** | Comparability of cohorts on the basis of the design or analysis | Study controls for potential confounders using propensity scores to compare outcomes between treatment arms |
| ***Outcome*** | Assessment of outcome | Record linkage |
|  | Was follow-up long enough for outcomes to occur | Yes |
|  | Adequacy of follow up of cohorts | Subjects lost to follow up unlikely to introduce bias (10%) - small number lost |
| ***Method of adjustment*** | - | Propensity score matching |
| **Yuan Z et al.** |  |  |
| ***Selection*** | Representativeness of the exposed cohort | Somewhat representative |
|  | Selection of the non-exposed cohort | Drawn from the same community as the exposed cohort |
|  | Ascertainment of exposure | Secure record (e.g., surgical records) |
|  | Demonstration that outcome of interest was not present at start of study | Yes |
| ***Comparability*** | Comparability of cohorts on the basis of the design or analysis | Study controls for potential confounders using propensity scores to compare outcomes between treatment arms |
| ***Outcome*** | Assessment of outcome | Record linkage |
|  | Was follow-up long enough for outcomes to occur | Yes |
|  | Adequacy of follow up of cohorts | Subjects lost to follow up unlikely to introduce bias (10%) - small number lost |
| ***Method of adjustment*** | - | Propensity score matching |
| **Udell JA et al.** |  |  |
| ***Selection*** | Representativeness of the exposed cohort | Somewhat representative |
|  | Selection of the non-exposed cohort | Drawn from the same community as the exposed cohort |
|  | Ascertainment of exposure | Secure record (e.g., surgical records) |
|  | Demonstration that outcome of interest was not present at start of study | Yes |
| ***Comparability*** | Comparability of cohorts on the basis of the design or analysis | Study controls for potential confounders using propensity scores to compare outcomes between treatment arms |
| ***Outcome*** | Assessment of outcome | Record linkage |
|  | Was follow-up long enough for outcomes to occur | Yes |
|  | Adequacy of follow up of cohorts | Subjects lost to follow up unlikely to introduce bias (10%) - small number lost |
| ***Method of adjustment*** | - | Propensity score matching |
| **McGurnaghan SJ et al.** |  |  |
| ***Selection*** | Representativeness of the exposed cohort | Truly representative |
|  | Selection of the non-exposed cohort | Drawn from the same community as the exposed cohort |
|  | Ascertainment of exposure | Secure record (e.g., surgical records) |
|  | Demonstration that outcome of interest was not present at start of study | No |
| ***Comparability*** | Comparability of cohorts on the basis of the design or analysis | Not clear |
| ***Outcome*** | Assessment of outcome | Record linkage |
|  | Was follow-up long enough for outcomes to occur | Yes |
|  | Adequacy of follow up of cohorts | Subjects lost to follow up unlikely to introduce bias (10%) - small number lost |
| ***Method of adjustment*** | - | None |
| **Sung J et al.** |  |  |
| ***Selection*** | Representativeness of the exposed cohort | Somewhat representative |
|  | Selection of the non-exposed cohort | Drawn from the same community as the exposed cohort |
|  | Ascertainment of exposure | Secure record (e.g., surgical records) |
|  | Demonstration that outcome of interest was not present at start of study | No |
| ***Comparability*** | Comparability of cohorts on the basis of the design or analysis | Study controls for an additional factor (e.g., cardiovascular disease) |
| ***Outcome*** | Assessment of outcome | Record linkage |
|  | Was follow-up long enough for outcomes to occur | Yes |
|  | Adequacy of follow up of cohorts | Complete follow up - all subjects accounted for |
| ***Method of adjustment*** | - | Matched by age, duration of diabetes, HbA1c, and smoking status |
| **Ueda P et al.** |  |  |
| ***Selection*** | Representativeness of the exposed cohort | Truly representative |
|  | Selection of the non-exposed cohort | Drawn from the same community as the exposed cohort |
|  | Ascertainment of exposure | Secure record (e.g., surgical records) |
|  | Demonstration that outcome of interest was not present at start of study | No |
| ***Comparability*** | Comparability of cohorts on the basis of the design or analysis | Study controls for potential confounders using propensity scores to compare outcomes between treatment arms |
| ***Outcome*** | Assessment of outcome | Record linkage |
|  | Was follow-up long enough for outcomes to occur | Yes |
|  | Adequacy of follow up of cohorts | Subjects lost to follow up unlikely to introduce bias (10%) - small number lost |
| ***Method of adjustment*** | - | Propensity score matching |
| **Woo V et al.** |  |  |
| ***Selection*** | Representativeness of the exposed cohort | Somewhat representative |
|  | Selection of the non-exposed cohort | Not clear |
|  | Ascertainment of exposure | Secure record (e.g., surgical records) |
|  | Demonstration that outcome of interest was not present at start of study | No |
| ***Comparability*** | Comparability of cohorts on the basis of the design or analysis | Not clear |
| ***Outcome*** | Assessment of outcome | Independent blind assessment |
|  | Was follow-up long enough for outcomes to occur | Yes |
|  | Adequacy of follow up of cohorts | No statement |
| ***Method of adjustment*** | - | N/A |
| **Yang JY et al.** |  |  |
| ***Selection*** | Representativeness of the exposed cohort | Somewhat representative |
|  | Selection of the non-exposed cohort | Drawn from the same community as the exposed cohort |
|  | Ascertainment of exposure | Secure record (e.g., surgical records) |
|  | Demonstration that outcome of interest was not present at start of study | Yes |
| ***Comparability*** | Comparability of cohorts on the basis of the design or analysis | Study controls for potential confounders using propensity scores to compare outcomes between treatment arms |
| ***Outcome*** | Assessment of outcome | Record linkage |
|  | Was follow-up long enough for outcomes to occur | Yes |
|  | Adequacy of follow up of cohorts | Subjects lost to follow up unlikely to introduce bias (10%) - small number lost |
| ***Method of adjustment*** | - | Propensity score matching |
| **Fralick M et al.** |  |  |
| ***Selection*** | Representativeness of the exposed cohort | Truly representative * |
|  | Selection of the non-exposed cohort | Drawn from the same community as the exposed cohort * |
|  | Ascertainment of exposure | Secure record (eg surgical records) * |
|  | Demonstration that outcome of interest was not present at start of study | No |
| ***Comparability*** | Comparability of cohorts on the basis of the design or analysis | Study controls for potential confounders using propensity scores to compare outcomes between treatment arms * |
| ***Outcome*** | Assessment of outcome | Record linkage * |
|  | Was follow-up long enough for outcomes to occur | Yes * |
|  | Adequacy of follow up of cohorts | Subjects lost to follow up unlikely to introduce bias (10%) - small number lost * |
| ***Method of adjustment*** | - | Propensity score matching |
| **Kaku K et al.** |  |  |
| ***Selection*** | Representativeness of the exposed cohort | Somewhat representative * |
|  | Selection of the non-exposed cohort | n/a |
|  | Ascertainment of exposure | Secure record (eg surgical records) * |
|  | Demonstration that outcome of interest was not present at start of study | No |
| ***Comparability*** | Comparability of cohorts on the basis of the design or analysis | n/a |
| ***Outcome*** | Assessment of outcome | Independent blind assessment * |
|  | Was follow-up long enough for outcomes to occur | Yes * |
|  | Adequacy of follow up of cohorts | Follow up rate <90% and no description of those lost |
| ***Method of adjustment*** | - | n/a |
| **Kashambwa R et al.** |  |  |
| ***Selection*** | Representativeness of the exposed cohort | Somewhat representative * |
|  | Selection of the non-exposed cohort | Drawn from the same community as the exposed cohort * |
|  | Ascertainment of exposure | Secure record (eg surgical records) * |
|  | Demonstration that outcome of interest was not present at start of study | No |
| ***Comparability*** | Comparability of cohorts on the basis of the design or analysis | Study controls for potential confounders using propensity scores to compare outcomes between treatment arms * |
| ***Outcome*** | Assessment of outcome | Record linkage * |
|  | Was follow-up long enough for outcomes to occur | Yes * |
|  | Adequacy of follow up of cohorts | Subjects lost to follow up unlikely to introduce bias (10%) - small number lost * |
| ***Method of adjustment*** | - | Propensity score matching |
| **Patorno E et al. (1)** |  |  |
| ***Selection*** | Representativeness of the exposed cohort | Somewhat representative * |
|  | Selection of the non-exposed cohort | Drawn from the same community as the exposed cohort * |
|  | Ascertainment of exposure | Secure record (eg surgical records) * |
|  | Demonstration that outcome of interest was not present at start of study | No |
| ***Comparability*** | Comparability of cohorts on the basis of the design or analysis | Study controls for potential confounders using propensity scores to compare outcomes between treatment arms * |
| ***Outcome*** | Assessment of outcome | Record linkage * |
|  | Was follow-up long enough for outcomes to occur | Yes * |
|  | Adequacy of follow up of cohorts | Subjects lost to follow up unlikely to introduce bias (10%) - small number lost * |
| ***Method of adjustment*** | - | Propensity score matching |
| **Patorno E et al. (2)** |  |  |
| ***Selection*** | Representativeness of the exposed cohort | Truly representative * |
|  | Selection of the non-exposed cohort | Drawn from the same community as the exposed cohort * |
|  | Ascertainment of exposure | Secure record (eg surgical records) * |
|  | Demonstration that outcome of interest was not present at start of study | No |
| ***Comparability*** | Comparability of cohorts on the basis of the design or analysis | Study controls for potential confounders using propensity scores to compare outcomes between treatment arms * |
| ***Outcome*** | Assessment of outcome | Record linkage * |
|  | Was follow-up long enough for outcomes to occur | Yes * |
|  | Adequacy of follow up of cohorts | Subjects lost to follow up unlikely to introduce bias (10%) - small number lost * |
| ***Method of adjustment*** | - | Propensity score matching |
| **Paul S et al.** |  |  |
| ***Selection*** | Representativeness of the exposed cohort | Truly representative * |
|  | Selection of the non-exposed cohort | Drawn from the same community as the exposed cohort * |
|  | Ascertainment of exposure | Secure record (eg surgical records) * |
|  | Demonstration that outcome of interest was not present at start of study | Yes * |
| ***Comparability*** | Comparability of cohorts on the basis of the design or analysis |  |
| ***Outcome*** | Assessment of outcome | Record linkage * |
|  | Was follow-up long enough for outcomes to occur | Yes * |
|  | Adequacy of follow up of cohorts | Subjects lost to follow up unlikely to introduce bias (10%) - small number lost * |
| ***Method of adjustment*** | - | n/a |
| **Belaez-Bejarano A et al.** |  |  |
| ***Selection*** | Representativeness of the exposed cohort | Truly representative * |
|  | Selection of the non-exposed cohort | Drawn from the same community as the exposed cohort * |
|  | Ascertainment of exposure | Secure record (eg surgical records) * |
|  | Demonstration that outcome of interest was not present at start of study | Yes * |
| ***Comparability*** | Comparability of cohorts on the basis of the design or analysis |  |
| ***Outcome*** | Assessment of outcome | Record linkage * |
|  | Was follow-up long enough for outcomes to occur | Yes * |
|  | Adequacy of follow up of cohorts | Subjects lost to follow up unlikely to introduce bias (10%) - small number lost * |
| ***Method of adjustment*** | - | n/a |
| **Udell JA et al.** |  |  |
| ***Selection*** | Representativeness of the exposed cohort | Somewhat representative * |
|  | Selection of the non-exposed cohort | Drawn from the same community as the exposed cohort * |
|  | Ascertainment of exposure | Secure record (eg surgical records) * |
|  | Demonstration that outcome of interest was not present at start of study | Yes * |
| ***Comparability*** | Comparability of cohorts on the basis of the design or analysis | Study controls for potential confounders using propensity scores to compare outcomes between treatment arms * |
| ***Outcome*** | Assessment of outcome | Record linkage * |
|  | Was follow-up long enough for outcomes to occur | Yes * |
|  | Adequacy of follow up of cohorts | Subjects lost to follow up unlikely to introduce bias (10%) - small number lost * |
| ***Method of adjustment*** | - | Propensity score matching |
